# Miss Elizabeth Blackwell, M. D.

**Published:** 1849-02

**Authors:** 


*Miss Elizabeth Blackwell, M. D.*—This lady, having attended two full courses of lectures at Geneva Medical College, and passed a satisfactory examination, received the degree of M. D. on the 23d ult. This, so far as we know, is the first instance of a female graduating at a medical college in this country. Miss B. was refused admission as a pupil at several medical colleges. Her inaugural thesis, as the reader will perceive, is contained in the original department of this number of our Journal.

